# Treatment options for persistent lateral femoral cutaneous nerve lesions after total hip arthroplasty via the direct anterior approach: retrospective analysis with clinical assessment

**DOI:** 10.1007/s00264-025-06512-5

**Published:** 2025-03-26

**Authors:** Jakob Hax, Louis Leuthard, Selina Nauer, Vincent A. Stadelmann, Michael Leunig, Hannes A. Rüdiger

**Affiliations:** 1 Schulthess-Klinik, Zurich, Switzerland; 2 University of Zurich, Zurich, Switzerland

**Keywords:** Complication, Direct anterior approach, Lateral femoral cutaneous nerve, Nerve injury, Total hip arthroplasty

## Abstract

**Purpose:**

The direct anterior approach (DAA) in total hip arthroplasty (THA) has a risk of lateral femoral cutaneous nerve (LFCN) injury. Long-term outcomes and therapeutic options for such injuries are poorly investigated. This study evaluates the impact of iatrogenic LFCN lesions on long-term outcomes and investigates treatments like ultrasound-guided nerve infiltration or neurolysis.

**Methods:**

Our institutional database of primary THAs (2014–2022) was searched for patients with iatrogenic LFCN lesions after DAA, confirmed via ultrasound or electroneurography. First, retrospective analysis of the effects of ultrasound-guided nerve infiltration and neurolysis. Second, clinical-radiological assessment of pain, function, incision, and affected skin area. Patient-reported outcomes (OHS, COMI Hip, UCLA) were compared to a matched non-LFCN injury control group.

**Results:**

Of 8136 patients, 29 (0.36%) met inclusion criteria, with 22 undergoing ultrasound diagnostics. Eighteen received nerve infiltration (improvement after one (n = 7), two (n = 3) or three (n = 1) infiltrations), and two had neurolysis. After a mean follow-up of 4.95 years, 13 patients were assessed. Common symptoms included hypesthaesia (11), dysesthesia (6), and tingling (3), with a mean affected area of 253cm^2^ ± 64.8. 24-months questionnaires for the LFCN group (OHS 39.2 ± 8.6, COMI Hip 2.4 ± 2.7, UCLA 6.5 ± 1.6) were worse than the control group (OHS 46.2 ± 2.3, COMI Hip 0.6 ± 0.8, UCLA 7.2 ± 1.5), though differences were not statistically significant.

**Conclusion:**

Instrumentally proven LFCN lesions after DAA THA are rare but lead to worse long-term outcomes. Ultrasound-guided nerve infiltration shows favorable results for symptom management.

## Introduction

The direct anterior approach (DAA) for total hip arthroplasty (THA) has shown its potential for faster postoperative recovery, improved functional outcomes, reduced soft tissue damage and lower dislocation rates [1, 2]. It is the only approach to the hip in a true intervascular and internervous plane [3, 4]. Despite these advantages, the DAA is associated with a risk of lateral femoral cutaneous nerve (LFCN) injury [5, 6]. The LFCN originates from the second and third lumbar nerve roots and supplies sensation to the skin of the antero-lateral thigh without any motor function. The course on the proximal thigh is very variable but runs in most cases closer to the DAA interval than to the anterolateral approach, making it more susceptible to injury when the DAA is used [7]. An injury to the LFCN results mostly in temporary numbness or paresthesia but may provoke chronic pain in the antero-lateral thigh [8]. Ultrasound-guided nerve infiltration has shown a positive effect on idiopathic meralgia paresthetica [9], however the effect after an iatrogenic postoperative lesion remains unclear. While such injuries have been described in previous studies [8, 10], there is a lack of data on the long-term course of objectively diagnosed postoperative LFCN lesions.

This study aims to evaluate the effect of persistent LFCN symptoms after primary THA using the DAA on long-term outcomes in terms of patient-reported outcome measures (PROMs), pain and sensory evaluation, as well as to describe the effect of treatment modalities like ultrasound-guided nerve infiltration and surgical neurolysis. Furthermore, this study compares patients with LFCN-injuries with a matched THA control group (cTHA) without LFCN symptoms regarding clinical and radiological outcomes. We hypothesize that such objectively confirmed lesions are rare yet have relevant impact on the long-term clinical course.

## Materials and Methods

### Study design and patient selection

**Fig. 1:**
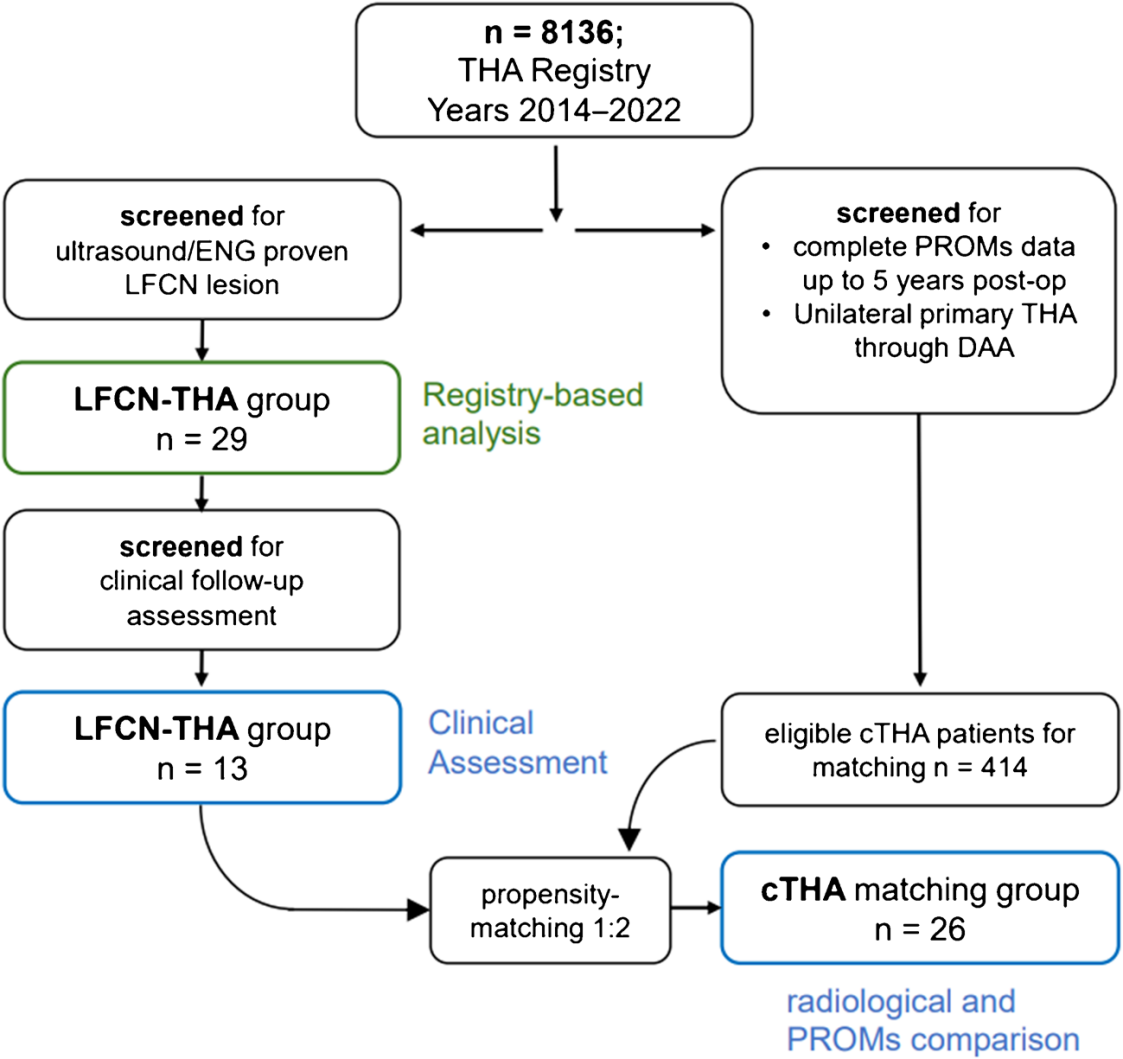
Flowchart of patient selection

A single-centre retrospective study was performed including patients who received primary THA through a DAA between 2014 and 2022 at our clinic and suffered from a postoperative LFCN injury confirmed by a neurologist using ultrasound diagnostics or additionally electroneurography (ENG). Patients who underwent revision surgery after the index operation, as well as those with previous surgeries on the affected hip, preoperative or bilateral LFCN symptoms, lumboradicular symptoms or concomitant interventions were excluded (Fig. 1). Out of 8136 THA screened from the institutional THA registry, 29 eligible LFCN patients were identified and were retrospectively evaluated.

For further analysis, these patients were invited for a clinical assessment with additional radiological measurements. 13 patients consented to participate in the clinical assessment of the study.

Finally, the PROMs of these patients were compared to those of the matching cTHA group without any LFCN symptoms. A pool of eligible patients was identified from the THA registry based on the following criteria: unilateral primary THA through DAA and complete PROMs at baseline, six, 12-, 24- and 60-months postoperative. From an eligible pool of 414 patients, a 1:2 propensity score matching was performed according to age, sex, body mass index (BMI) and American Society of Anesthesiologists (ASA) classification to compare the 13 LFCN patients to 26 matching cTHA patients without LFCN injury.

Baseline parameters were documented for all patients, including sociodemographics, general health status, and anamnesis. This study was approved by the local ethical committee of Zurich (KEK-ZH-Nr. 2023-01287), all participants provided informed consent.

### Surgical procedure and Rehabilitation

All surgical procedures were performed by high-volume hip surgeons (> 200 THA /year) under spinal or total anaesthesia. Surgery was performed in supine position on a regular operating table (i.e. without traction device of leg holder). Oblique (Bikini) or traditional longitudinal skin incisions were performed as we have previously described in detail [11]. The classical longitudinal incision started slightly lateral of the anterosuperior iliac spine (ASIS) and were directed in line with the fibres of the tensor fascia latae muscle (TFL). The Bikini incision was placed slightly distal to the inguinal crease and mostly lateral to the ASIS as we have previously described [11]. In both cases, the subcutaneous dissection is performed in a blunt fashion and the fascia of the TFL was incised in line with its fibers. Deep dissection was performed within the TFL fascial sheet to prevent direct damage to the LFCN. Intervals between the sartorius and TFL and the rectus femoris and TFL, respectively, were prepared by blunt dissection. The ascending branches of the lateral femoral circumflex artery were ligated or coagulated. The hip joint capsule was then exposed and opened as previously described [11]. Subsequently, THA was performed [11, 12]. Intraoperative periarticular infiltration using 1 ml Ketorolac 30 mg/ml and 3 ml Lidocain 1% was performed as standard. The TFL fascia was closed with a running absorbable suture (Ethicon Vicryl 2–0, Johnson & Johnson, New Brunswick, USA). Subcutaneous tissue was closed with interrupted absorbable stitches (Ethicon Vicryl 2–0). The skin was closed with an intracutaneous absorbable running suture (Ethicon Stratafix 3–0). All patients followed an identical postoperative rehabilitation protocol including full weight-bearing at the day of surgery.

### Neurological consultation

All patients with a suspected LFCN lesion at the surgical follow-up were referred to a standardized neurological examination by a board-certified neurologist. Ultrasound diagnostics or additional ENG were used to identify new postoperative LFCN lesion. The diagnosis was confirmed when a neuroma or typical caliber jump was detected in the ultrasound. In case of ENG, sensory nerve action potentials (SNAP) were assessed to evaluate the function of the sensory nerve. LFCN lesions may decrease the amplitude of the SNAP, indicating damage to the sensory fibres of the nerve [13, 14]. In case of predominantly painful symptoms (dysesthesia) or tingling paraesthesia, a diagnostic-therapeutic ultrasound-guided nerve infiltration was performed using 3 ml Rapidocaine 1% and 1 ml of methylprednisolone acetate (40 mg/ml). This was standardized and carried out by the same board-certified neurologist with ultrasound-guided flooding of the LFCN about 1 cm distal and lateral to the ASIS in the interval between the sartorius and tensor fasciae latae muscles under sterile conditions. The effect of the infiltration was routinely documented by phone call one day after the intervention and followed up clinically after three months. In cases of refractory pain after initial improvement, the infiltration was then repeated twice at maximum with at least a three month interval in between the interventions. In patients with less symptoms, medication with topical capsaicin lotion 0.1% or oral Pregabalin was prescribed. Patients with refractory symptoms were referred to the surgeon for surgical neurolysis.

### Registry-based analysis

The registry-based analysis included the evaluation of baseline parameters (sex, age, BMI, time of surgery, blood loss and smoking), documented symptoms (Visual Analog Scale pain from 0 = no pain to 10 = worst pain that I can imagine, hypaesthesia: yes/no, dysaesthesia: yes/no, tingling: yes/no, skin incision type: oblique/bikini or longitudinal), LFCN diagnostics (ultrasound and additional ENG) as well as the therapeutic treatment pathway including medication (Pregabalin, Capsaicin lotion), ultrasound-guided nerve infiltrations and neurolysis.

In addition, PROMs were collected using questionnaires (preoperative, 6, 12, 24 and 60 months postoperative) in paper or digital form, completed by the patients in their homes. The Core Outcome Measurement Index (COMI) hip [15], Oxford Hip Score (OHS) [16] and University of California at Los Angeles (UCLA) activity scale [17] were evaluated. The COMI-hip questionnaire is a 6-item instrument assessing pain, function, symptom-specific well-being, disability (work and social) and quality of life. The score ranges from 0 (best outcome) to 10 (worst outcome). The OHS, adapted and validated in German, is evaluating pain and disability in patients with a hip arthroplasty over the preceding four weeks with 12 different questions. The best score is indicated by a total sum score of 48, the worst by 0. The UCLA scale classifies the activity level from 1 (no physical activity) to 10 (regular participants in impact sports).

Infiltration outcomes were additionally analyzed by two single questions about general treatment outcome ("*How much did the treatment of your hip complaints help you overall? – helped a lot, helped, helped only a little, didn't help, made things worse")* and satisfaction ("*How satisfied are you with the treatment of your hip complaints at our clinic so far*? *– very satisfied, somewhat satisfied, neither satisfied nor dissatisfied, somewhat dissatisfied, very dissatisfied").* The answers of the closest timepoint after infiltration date were used, but with a maximum of six months range post-intervention.

### Clinical assessment

**Fig. 2:**
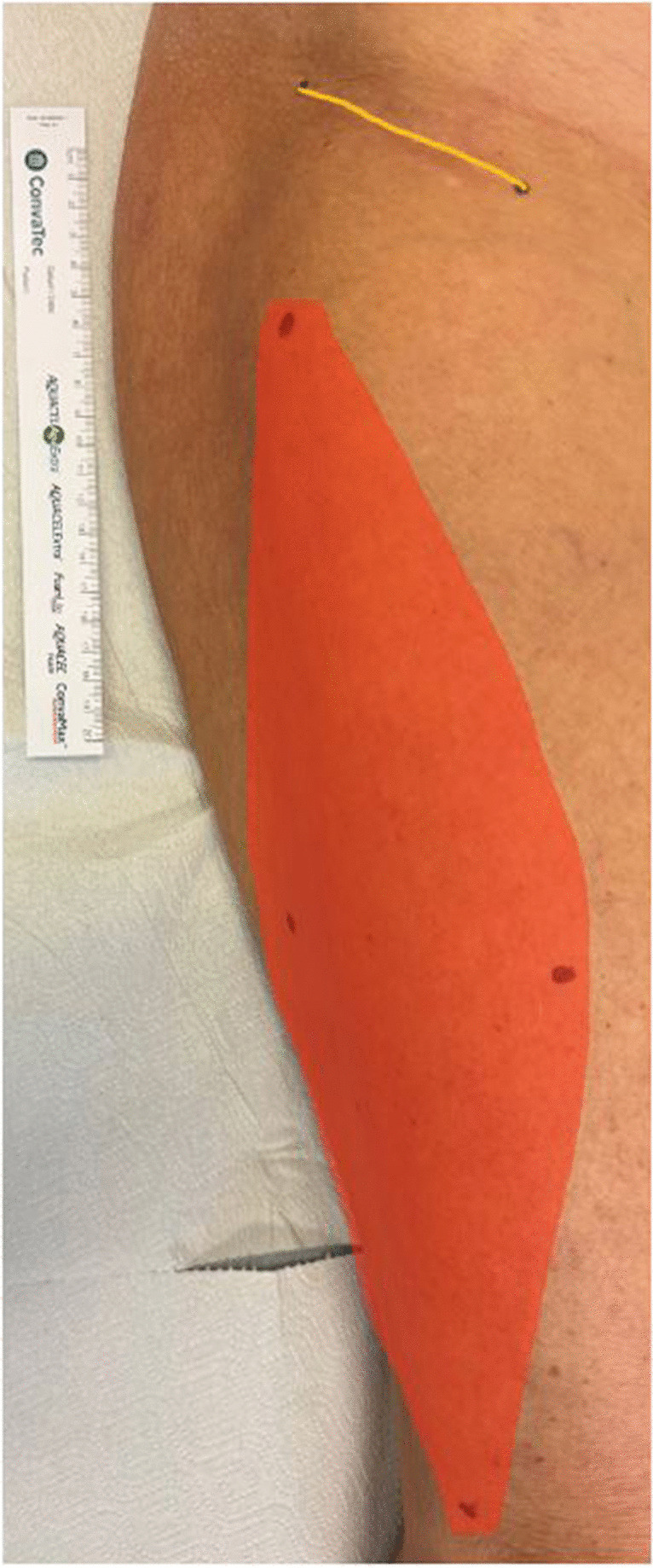
Right hip with upper thigh; Example of the marked scar (yellow line) and the affected skin area (orange) including measurement during the clinical follow-up assessment

All clinical follow-up assessments were performed by a board-certified surgeon (XX). In addition to a standard orthopaedic assessment, neurological deficits, VAS pain score and specific features related to the LFCN injury were assessed. Muscular deficits were reported according to the Medical Research Council (MRC) Scale [18]. Neurological deficits were assessed by the Trendelenburg sign, the Lasègue test and the Tinel sign. The Trendelenburg sign was considered positive if the patient could not maintain a single leg stance for at least 30 s. [19]. The Lasègue test was considered positive if the angle to which the leg can be raised (upon straight leg raising) is < 45° before eliciting pain [20]. The Tinel sign was considered positive if the LFCN is lightly tapped proximally and the patient reported paraesthesia or pain radiating distally into the sensory nerve supply area [21]. The following symptoms specific to LFCN were measured: hypaesthesia, dysaesthesia, and tingling, size of the affected skin area (length and width), type of incision (longitudinal, oblique), length of the skin incision, and distance from it to the ASIS. The scar and the affected skin area were marked, and photo documented during the examination (see example Fig. 2).

All postoperative complications and reoperations were recorded (adverse events).

### Radiological Outcomes

Radiographs were assessed in a plain ap-pelvic view and a cross-table lateral view by experienced radiology technicians based on specific in-house protocols. The cup inclination [°], the stem position (varus, valgus, neutral), the radiological leg length difference (mm), signs of loosening or heterotopic ossifications were determined on digital radiographs (JiveX Diagnostic version 5.3.0.7, VISUS Health IT GmbH).

### Statistical analysis

Data was managed using the REDCap Electronic Data Capture system [22] and exported for statistical analysis in RStudio version 4.3.0 [23]. Standard descriptive statistics were used to summarize baseline and follow-up data included absolute and relative frequencies for categorical data and means and standard deviations for continuous data.

Propensity score matching was used to derive a matching cTHA group from the registry for comparison of outcomes with the eligible LFCN-THA group using the MatchIt R package [24]. This method computes a nearest neighbour distance between each treated and control unit and matches the control unit with the smallest distance to the treated unit. To increase the balance of the covariates in the control group, a matching ratio of 1:2 was used. Overall, this technique reduces confounding bias by distributing patient characteristics uniformly between treatment groups, thereby increasing the chance to indicate treatment effects retrospectively [24]. The following variables were matched: sex (male/female), age at surgery (years), BMI (kg/m2), and general health status based on ASA (grades 1/2/3). Differences in radiographic measurements and clinical follow-up assessments between LFCN-THA and cTHA patients were evaluated using two‐sample t test or Wilcoxon rank‐sum test as appropriate. Comparative analyses of PROMs were made using t test with Bonferroni correction between groups at different time points.

## Results

### Registry-based analysis

Out of 8136 patients enrolled in the institutional THA registry between 2014 and 2022, 29 (0.36%; 7 surgeons) met the inclusion criteria with a confirmed iatrogenic LCFN lesion. An oblique (bikini) incision was performed in 16 patients and a longitudinal incision in 13. A total of 26 patients reported persistent pain in the skin area supplied by the LFCN, ten reported hyposensitivity, and seven patients had combined symptoms. Ultrasound diagnostics alone was performed in 22 cases and an additional ENG examination in seven cases.

Out of the 29 LFCN patients, 18 patients received one or multiple ultrasound-guided nerve infiltration, eight patients had self-limiting symptoms without further therapy, one patient underwent directly surgical neurolysis, and only two received pharmacological therapy.

**Fig. 3:**
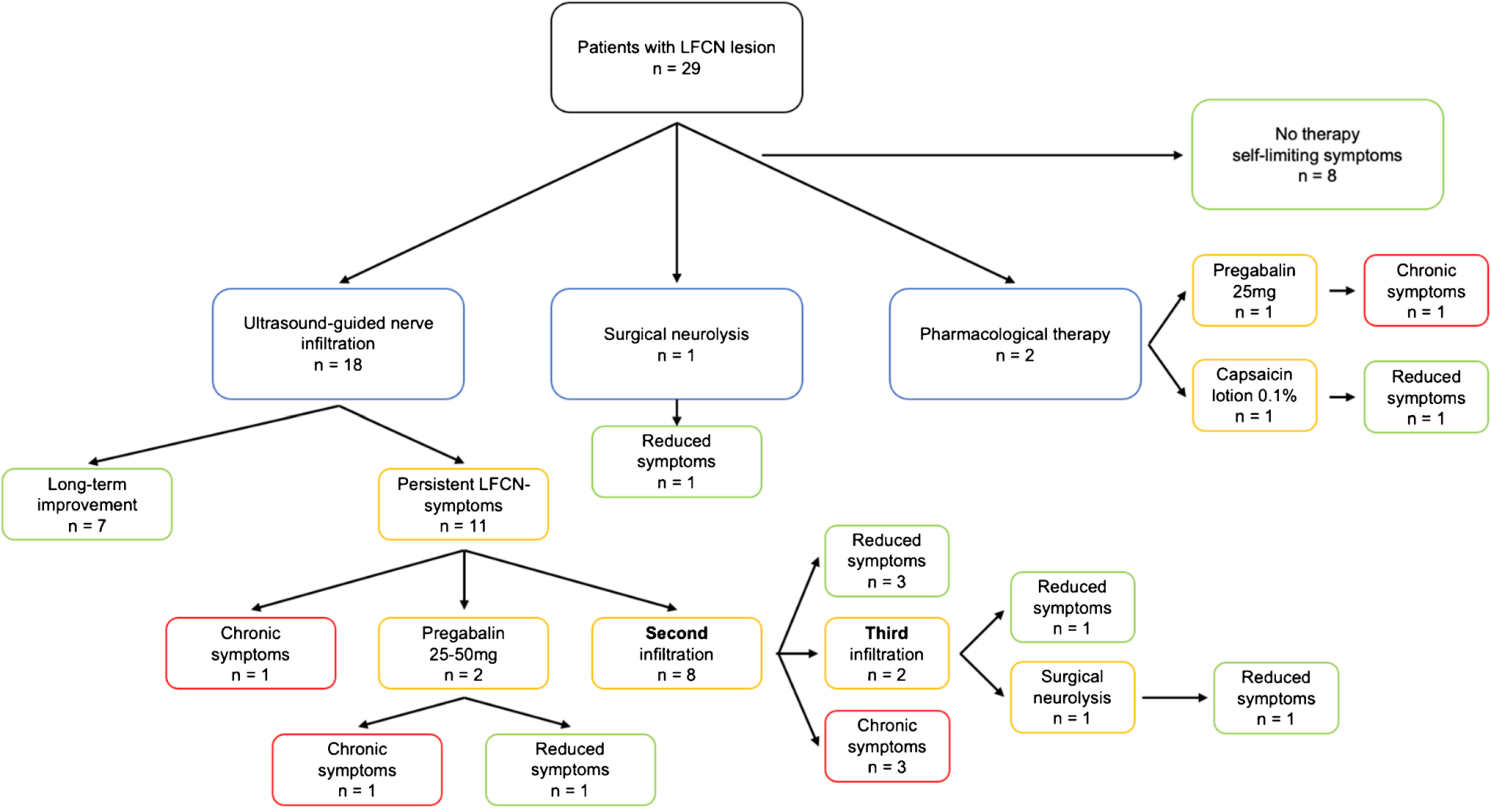
Flowchart of therapeutic interventions and outcomes of patients included in the registry-based analysis. All Lateral Femoral Cutaneous Nerve (LFCN)-THA patients, black box. Therapeutic interventions, blue boxes. Patients with persistent symptoms and still under treatment, yellow boxes. Patients with completed treatment and clinically relevant improved symptoms, green boxes. Patients with completed treatment and chronic, persistent symptoms, red boxes. mg, milligram

After the first infiltration, six out of ten patients answered the general treatment outcome questions with "*helped a lot*" or "*helped*", while 2 and 1 reported "*didn't help*" or "*helped only a little*" respectively. After the second infiltration, one patient answered "*helped a lot*", one "*helped*", one "*helped only a little*" and one "*didn't help*". One patient who received three infiltrations answered "*made things worse*" after all infiltrations. A total of seven out of 18 infiltrated patients benefited immediately from the first infiltration and did not require any additional therapy over the observation period of 60 months. Another four patients achieved sustained benefit from the second and third infiltration without the need for a further intervention. In the analyzed postoperative course of the collective, chronic LFCN symptoms were documented in the medical records of six of the 29 patients (Fig. 3).

**Fig. 4:**
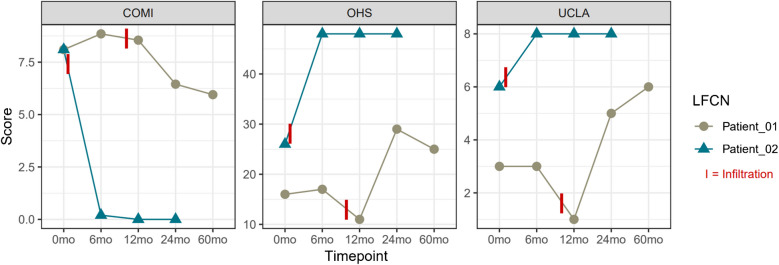
Development of the COMI hip, OHS and UCLA scores after surgical neurolysis in Patient_01 (brown) 10 months and in Patient_02 (blue) 6 days postoperatively

**Fig. 5:**
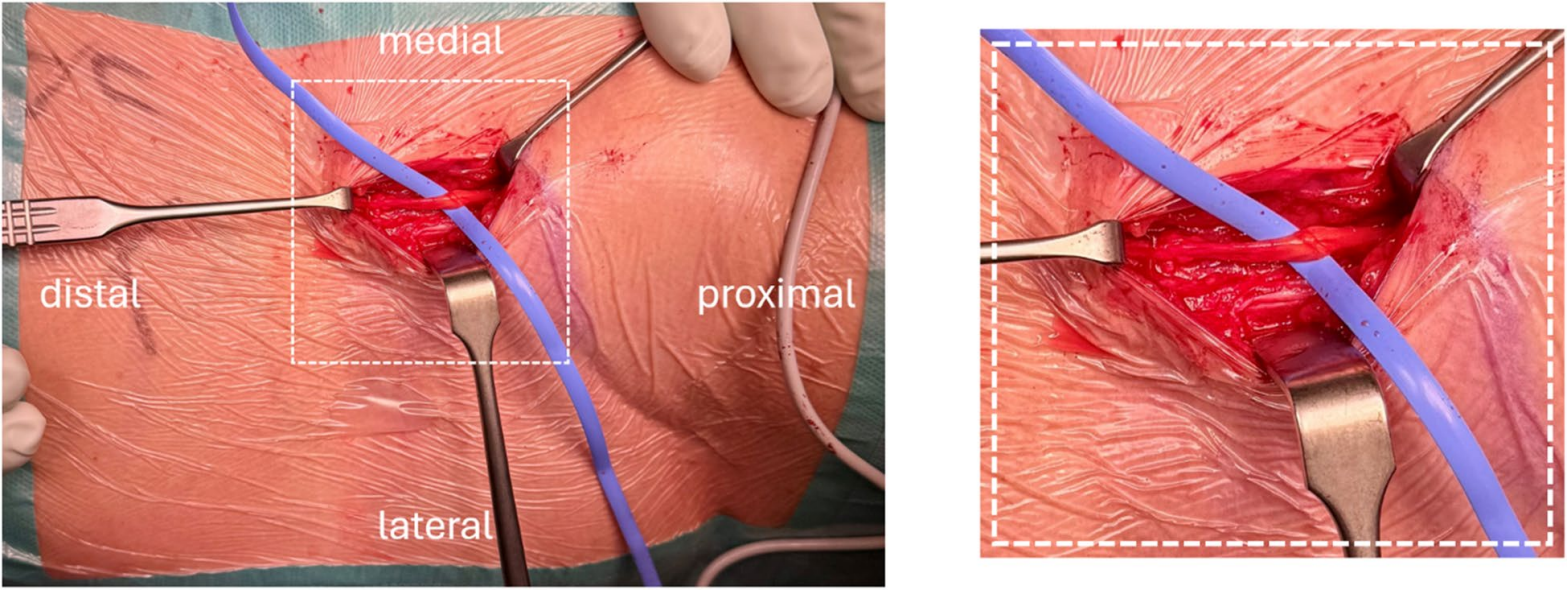
Lateral femoral cutaneous nerve after neurolysis in the course of the longitudinal incision on the proximal thigh immediately distal to the anterior superior iliac spine. Thickening of the nerve with distally altered nerve structure can be seen

Two patients underwent surgical neurolysis: the first one (Patient_01, Fig. 4) with a suspected sutured nerve in the fascia of the TFL received a revision with surgical neurolysis on postop day six. The second patient (Patient_02, Fig. 4) underwent surgical neurolysis with decompression of the LFCN from the iliac crest well distally (length 10 cm, Fig. 5) and relocation of the lateral branch ten months postoperatively for refractory pain after three ultrasound-guided nerve infiltrations, each with short-term response only. Both patients showed an improvement in PROMs following neurolysis.

### Clinical assessment

A detailed overview of patient characteristics and perioperative details of the 13 LFCN patients participating in the clinical assessment and their matched control group cTHA is shown in Table 1. No difference in any characteristic could be identified. Since the patients of the LFCN-THA group underwent surgery at different time points, an average follow-up time of 4.95 ± 1.92 years was obtained for the clinica-radiological study assessment.

**Table 1:** Clinical assessment and matched-pair cohort

	LFCN-THA(*n* = 13)^1^	cTHA(*n* = 26)^1^	*p*-value^2^
Baseline characteristics			
Female sex	8	13	0.496
Age [years]	60 ± 11	60 ± 11	0.905
BMI [kg/m^2^]	26.9 ± 3.6	27.5 ± 4.3	0.612
ASA class			0.712
1	1	2	
2	9	21	
3	3	3	
Symptom duration before THA [days]	42 ± 56	36 ± 52	0.664
*Missing*	*4*	*0*	
LOS [days]	4.9 ± 0.9	5.0 ± 1.9	0.842
Surgery duration [minutes]	70 ± 17	70 ± 12	0.754
Blood loss [ml]	268 ± 64	258 ± 107	0.432
*Missing*	*2*	*0*	
Skin incision			0.367
Oblique/Bikini	7	14	
Longitudinal	6	12	

^1^Data displayed as number or mean ± standard deviation; ^2^Wilcoxon rank sum test, Fisher’s exact test; *ASA class* American Society of Anesthesiologists classification, *BMI* Body Mass Index, *cTHA* control group Total Hip Arthroplasty, *LFCN-THA* Lateral Femoral Cutaneous Nerve – Total Hip Arthroplasty, *LOS* Length of stay in hospital

In the clinical follow-up collective (13 patients), 11 patients (85%) reported persistent hypesthesia in the LFCN skin area, six (46%) dysaesthesia and three (23%) tingling paraesthesia (multiple symptoms possible). Ultrasound-guided nerve infiltration was performed in 6 (46%). The mean length of the affected skin area was 23 ± 12 cm and width 11.0 ± 5.4 cm (average area 253 ± 64.8 cm^2^). Mean length of the scar was 9.15 ± 1.05 cm, with a mean distance from the ASIS of 4.38 ± 1.24 cm.

Clinical findings are summarized in Table 2 compared to the contralateral, non-operated side. No significant differences were detected between the two sides.

**Table 2:** Clinical follow-up assessment LFCN-THA

	**Operated side** **(n = 13)**^**1**^	**Non-operated side** **(n = 13)**^**1**^	**p-value**^**2**^
VAS Pain	2.00 ± 2.08	1.23 ± 2.49	0.2
ROM [°]			
Extension	10.00 ± 3.54	9.62 ± 3.20	0.8
Flexion	101 ± 6	100 ± 8	0.6
Inner rotation	24 ± 7	18 ± 11	0.1
Outer rotation	38.1 ± 3.3	34.6 ± 6.3	0.09
Abduction	37.7 ± 4.4	33.8 ± 7.1	0.1
Adduction	28 ± 9	24 ± 7	0.3
Trendelenburg sign positive	1	0	> 0.9
Lasegue sign positive	0	0	
Tinel sign positive	2	0	0.5
Hip flexion strength			0.2
M4	3	0	
M5	10	13	
Hip abduction strength			0.6
M4	4	2	
M5	9	11	

^1^Data displayed as number or mean ± standard deviation; ^2^Wilcoxon rank sum test, Fisher’s exact test; *LFCN‐THA* lateral femoral cutaneous nerve total hip arthroplasty, *ROM* range of motion, *VAS* Visual Analog Scale

Radiological follow-up results are shown in Table 3, also without significant group differences regarding cup inclination, socket alignment (neutral, varus, valgus), radiological leg length discrepancy and heterotopic ossifications. Patients in the cTHA group were only examined once radiologically around six weeks after surgery, resulting in a significantly shorter average follow-up time of 0.12 ± 0.01 years (p < 0.001).

**Table 3:** Radiological follow-up results

	LFCN-THA (*n* = 13)^1^	cTHA (*n* = 26)^1^	*p*-value^2^
Cup inclination [°]	43.9 ± 3.8	42.1 ± 2.9	0.13
Socket alignment			> 0.9
Neutral	9	18	
Varus	4	8	
Valgus	0	0	
Radiological leg length discrepancy [cm]	0.81 ± 2.84	0.46 ± 1.90	0.5
Heterotopic ossification	1	0	0.3

^1^Data displayed as number or mean ± standard deviation; ^2^Wilcoxon rank sum test, Fisher’s exact test; *cTHA* control group Total Hip Arthroplasty, *LFCN-THA* Lateral Femoral Cutaneous Nerve – Total Hip Arthroplasty

**Fig. 6:**
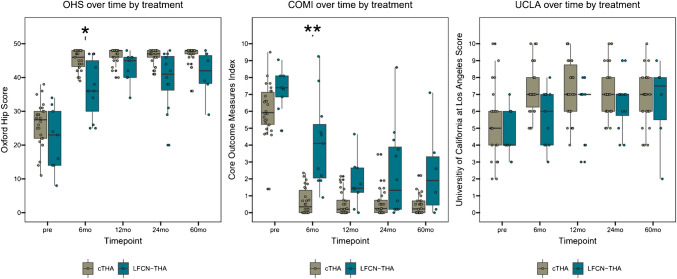
Box plots representing the Oxford Hip Score (OHS), Core Outcome Measures Index (COMI)‐hip and University of California at Los Angeles (UCLA) activity scale scores for LFCN-THA and cTHA patients at baseline and the 6‐, 12-, 24- and 60-month postoperative follow‐ups. Rectangle ends correspond to upper and lower quartiles of the data values. The line drawn through the rectangle corresponds to the median value. Whiskers, starting at the rectangle ends (or points representing extreme values), indicate minimum and maximum values. **p < 0.01 and *p < 0.05 mark significant differences between groups. Due to non-responders or missing/unanswered questionnaire items, there were 4, 2, 4, 1, 7 LFCN patients with missing PROMs at baseline, 6, 12, 24, 60 months respectively

The evolution of COMI hip, OHS and UCLA activity scale in comparison of LFCN-THA versus cTHA is shown in Fig. 6. There was a significant group difference in favour of the cTHA group for the COMI hip and OHS in the early phase six months postoperatively. Overall, the long-term results in the LFCN-THA group were worse up to 60 months postoperatively.

cTHA, control group Total Hip Arthroplasty; PROM, patient‐reported outcome measure; LFCN‐THA, lateral femoral cutaneous nerve total hip arthroplasty. In the clinical follow-up collective, there were no revisions or reoperations in either the LFCN-THA or the cTHA group. The cTHA group showed no surgery-specific complications. In the LFCN-THA group, the complications were limited to the iatrogenic LFCN lesions, six times with necessary intervention of ultrasound-guided nerve infiltration.

## Discussion

This study was conducted to investigate the effect of iatrogenic objectively proven LFCN lesions during primary THA using the DAA. First, affected patients were evaluated regarding symptoms and outcomes after treatment with ultrasound-guided nerve infiltration and surgical neurolysis. Second, available patients were clinically examined and compared to a matched THA control group without LFCN symptoms regarding PROMs and surgical data.

We identified 29 out of 8136 patients (0.36%) with a painful objectively proven LFCN lesion from our institutional THA registry over a period of eight years, suggesting that these lesions are rare in a high-volume surgical setting (> 200 THA/surgeon/year). However, we did not systematically evaluate all patients for persistent LFCN symptoms during follow-up assessments. Only patients with symptoms strong enough to trigger a specialized neurological examination were included. The incidence of symptomatic chronic lesions may hence be underestimated in this study.

Similar to our previously described collective [11], no significant difference in the occurrence of an LFCN lesion with a longitudinal or oblique (bikini) incision was found. In the clinical analysis of Goulding et al. [6] using questionnaires, two-thirds of the patients reported symptoms in the skin area supplied by the LFCN. On the contrary, Homma et al. [8] identified the LFCN lesion patients only based on their complaints defined as the presence of symptoms over the lateral aspect of the thigh excluding the operative scar area. This inaccuracy in diagnostic methods is also confirmed by our previous analysis [11], which found a large discrepancy between patient reported (20%) and objectively examined (60%) LFCN lesions, and may explain the contradictory reports of incidences of LFCN lesions after THA using the DAA [6, 8, 10, 25–27]. To objectively identify LFCN lesions in a reliable and accurate way, ultrasound diagnostics and ENG were used in the present analysis. The combination of these techniques allowed to confirm the diagnosis [14]. Good correlation was found between abnormal SNAP results in ENG and impairment of the LFCN [13].

Another important finding of the present study is the high number of patients (26 of 29) with chronic long-term pain in the skin area supplied by the LFCN, compared to less frequent hyposensitivities (10 of 29). Chronic, therapy-refractory symptoms were observed in six of 29 patients. This may explain the impaired short- and long-term PROM scores in the LFCN-THA compared to the cTHA group. Thus, an iatrogenic LFCN lesion can be considered a relevant and long-term impairing complication.

In another study, postoperative LFCN lesions were associated primarily with hypaesthesia and tingling, rather than pain [8]. Nevertheless, the symptoms did not improve independently in almost half of the cases, with also long-term impact on the clinical outcome and lower self-reported satisfaction score for postoperative quality of life. In the same study, the Forgotten Joint Scale (FJS-12) also showed significantly worse values in patients with LFCN lesion compared to non-LFCN injury THAs, although no difference could be seen in the Harris Hip Score (HHS) or Western Ontario and McMaster Universities Osteoarthritis Index (WOMAC). Similar to the current results, this is only partially comparable to the results of Bhargava et al. [10], who described mainly complete and self-limiting symptoms after LFCN lesions, with no impact on the functional outcome regarding the HHS.

The difference of the clinically relevant lower score values of LFCN-THA compared to cTHA patients is likely because we only included patients with a specialized neurological exam, who have more relevant symptoms including neuropathic pain and not just impairment of skin sensitivity. Self-limiting and clinically less relevant cases were not included in this study, so that only the negative effect of long-term limiting LFCN lesions could be shown. This effect seems to be underestimated in studies with equally included temporary and chronic LFCN symptoms.

This is the first study demonstrating long-term clinical improvement of ultrasound-guided nerve infiltration in case of iatrogenic postoperative neuropathic pain after LFCN lesions with seven of 18 patients already benefiting from the first, another three from the second and one more from the third infiltration (overall long-term clinical improvement in 11 of 18 patients). Persistent meralgia paraesthetica was the most common reason for infiltration, whereby it improved consistently and sustainably in most cases over the 60-month follow-up period according to the general treatment outcome questions analyzed. This is in line with the study of Tagliafico et al. [9], where ultrasound-guided infiltrations in 20 consecutive patients led to a significant improvement in pain after at least two months. Comparable to the present study, the infiltration had to be repeated in four patients with improvement of symptoms in all cases. Because both studies have investigated small cohorts, the benefits of a second infiltration remain unclear and larger prospective randomized studies on ultrasound-guided nerve infiltration in iatrogenic LFCN lesions after THA are needed. Generally, infiltration should be a first diagnostic-therapeutic option for patients with persistent symptoms. The further course depends on the intensity and duration of the positive effect of the treatment, whereby infiltration can be repeated in case of refractory symptoms with an initially good effect, although neurolysis should be evaluated if the symptoms persist. It is important that the infiltrations are carried out by the same experienced examiner in a reproducible and standardized manner using the same technique. To ensure comparability, this was considered in the present study.

Two patients with persistent severe pain needed surgical neurolysis as intervention. One patient was operated already six days after primary THA and improved in symptoms immediately postoperatively, followed by complete recovery. The other patient underwent surgical neurolysis ten months after primary THA with an immediate postoperative improvement in PROMs, yet with chronic pain and thus without complete recovery. Although the therapeutic effect of neurolysis is rather studied on the idiopathic type [28–30] than on iatrogenic LFCN lesions, it seems that the outcome of neurolysis depends on the preoperative duration of symptoms [29]. This is consistent with the results of the present study, assuming that long-lasting chronic symptoms hinder the full recovery of the nerve. The available data indicate that there are two groups of patients who require and may benefit from surgical neurolysis: On the one hand, patients who cannot be mobilized immediately postoperatively due to severe pain in the LFCN area due to nerve traction or entrapment (e.g. in a suture), and on the other hand, patients with chronic pain and failure of conservative treatment after repeated infiltrations.

Preventing iatrogenic LFCN lesions is essential, as they can lead to chronic pain and long-term impairment of quality of life. A possible strategy could be for example to shorten the skin incision proximally [7]. In addition, the operative technique should be adapted to avoid direct or indirect injury to the LFCN (e.g. pressure due to retractor placement). Furthermore, clinical symptoms of an LFCN lesion should be carefully evaluated during the first postoperative follow-up examination after THA, and the patient should be referred to further objective instrumental diagnostics in case of suspicious clinical presentation. Symptoms caused by an iatrogenic LFCN lesion should be recognized and treated as quickly as possible, to avoid chronification. Conservative treatment with ultrasound-guided nerve infiltrations should be the first step in most cases. For severe, therapy-refractory symptoms, surgical neurolysis should be considered, although the long-term benefit for long-lasting symptoms appears to be worse.

The current study has limitations. Due to the retrospective design without standardized clinical follow-up examinations, only a descriptive evaluation of the prospectively collected registry data was possible. In addition, the incidence of LFCN lesions is probably underestimated because short-term self-limiting LFCN lesions with few clinical symptoms and thus without referral to a neurologist for further instrumental diagnostics were not included to keep the cohort homogenous. Only patients who had in-house neurological examination have been included, which can cause a possible selection bias. Furthermore, less than half of the LFCN-THA patients were available for clinical-radiological follow-up examination. Thereby, the radiological follow-ups in the LFCN-THA group were performed at different time points compared to the cTHA group. Included were cases from seven different surgeons operating according to the same in-house standard, but it may also have an influence on the occurrence of an LFCN lesion. The statistical analysis for PROMs comparison is limited to simple t tests with Bonferroni correction due to incomplete or inconsistent data sets in the LFCN group. This approach was used for its simplicity and suitability for timepoint comparisons, despite the limited power of the Bonferroni correction detecting real differences in small sample sizes. Finally, no therapeutic recommendation or statement can be formulated about the investigated interventions due to the small sample size and the lack of a control group. However, it can be stated that the measures mentioned have mostly improved the symptoms caused by the LFCN lesion in the long term and thus generally represent valid options.

In conclusion, iatrogenic LFCN lesions confirmed by a neurologist after primary THA via the DAA are rare in a high-volume orthopaedic setting (> 200 THA/surgeon/year) and appear to be independent of the surgical incision technique (oblique/bikini vs. longitudinal). The clinical long-term course is negatively influenced by painful symptoms, yet ultrasound-guided nerve infiltration can lead to complete and persistent symptom recovery. In chronic, therapy-refractory cases, neurolysis should be performed as soon as possible to ensure the best possible recovery of the nerve.

## Data Availability

No datasets were generated or analysed during the current study.
